# Indicators for Evaluating the Performance and Quality of Care of Ambulatory Care Nurses

**DOI:** 10.1155/2015/861239

**Published:** 2015-08-24

**Authors:** Joachim Rapin, Danielle D'Amour, Carl-Ardy Dubois

**Affiliations:** ^1^Vaud University Hospital Center, rue du Bugnon 21, 1011 Lausanne, Switzerland; ^2^Faculty of Nursing, University of Montreal, Centre-Ville Station, P.O. Box 6128, Montreal, QC, Canada H3C 3JT

## Abstract

The quality and safety of nursing care vary from one service to another. We have only very limited information on the quality and safety of nursing care in outpatient settings, an expanding area of practice. Our aim in this study was to make available, from the scientific literature, indicators potentially sensitive to nursing that can be used to evaluate the performance of nursing care in outpatient settings and to integrate those indicators into the theoretical framework of Dubois et al. (2013). We conducted a scoping review in three databases (CINAHL, MEDLINE, and EMBASE) and the bibliographies of selected articles. From a total of 116 articles, we selected 22. The results of our study not only enable that framework to be extended to ambulatory nursing care but also enhance it with the addition of five new indicators. Our work offers nurses and managers in ambulatory nursing units indicators potentially sensitive to nursing that can be used to evaluate performance. For researchers, it presents the current state of knowledge on this construct and a framework with theoretical foundations for future research in ambulatory settings. This work opens an unexplored field for further research.

## 1. Introduction

Currently there is very little evidence available on nursing outcomes in ambulatory care. It is becoming increasingly important, however, to document performance in this activity sector, which is experiencing rapid expansion due particularly to the shift toward ambulatory care, population aging, and greater prevalence of chronic illnesses. Demand for ambulatory nursing services is growing as hospital stays are shortened and patients are followed up in their communities [1]. These patients, who present multiple health problems that are not only physical but also cognitive, require more monitoring, care, and health education [2].

To meet the needs generated by these changes in health and social services, ambulatory nursing has developed a broader and more diverse offer of services that has resulted in an increase in activities. The number of nurses allocated to this sector continues to grow [3]. Generally speaking, the ambulatory sector encompasses outpatient services provided in university and regional hospitals as well as in clinics, including emergency rooms and telehealth services [4].

Nevertheless, performance measurement in ambulatory nursing has been significantly slow to develop, compared with acute care and long-term care nursing. Except for the American Nursing Association's literature review [5] on nursing-sensitive quality indicators, particularly in ambulatory care, and the recent report of the task force mandated by the AAACN to identify nursing-sensitive quality indicators in ambulatory services [6], there is little literature available on this topic. On one hand, there is not much consensus on what constitutes nursing-sensitive performance indicators [7, 8], and, on the other hand, ambulatory nursing services remain, for the most part, underevaluated [7].

Yet a certain number of problems have been identified related to care quality and safety in the ambulatory sector, such as inappropriate wait times for treatment, lack of care coordination [9], and the occurrence of adverse events [10–12].

This paper presents the results of a scoping review undertaken to identify indicators that are sensitive to ambulatory nursing. As noted by Doran et al. ([13], p. 10) nursing-sensitive indicators are those that are “relevant, based on nurses' scope and domain of practice, and for which there is empirical evidence linking nursing inputs and interventions to the outcome.”

## 2. Theoretical Framework

**Figure 1:**
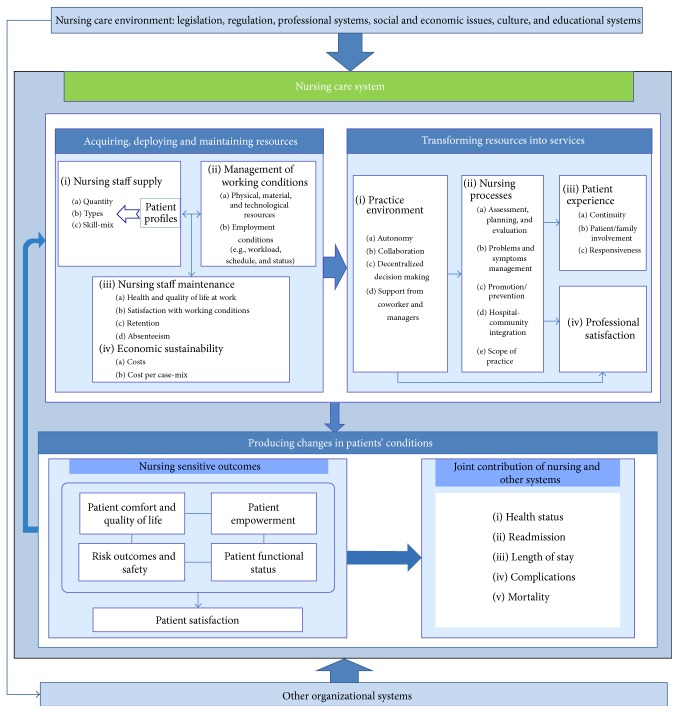
Nursing Care Performance Framework (NCPF) developed by Dubois et al. [15].

Some authors have promoted the development of theoretical frameworks specifically for ambulatory nursing performance [2, 14]. There are very few such frameworks, and each has significant theoretical and methodological shortcomings. In the absence of a robust and integrative framework, we used the Nursing Care Performance Framework (NCPF) developed by Dubois et al. [15] (Figure 1) to structure our study. Although the NCPF was developed largely based on acute care literature, its construction and rigour make it potentially applicable to the nursing ambulatory care context. The model includes three subsystems that operate together to achieve three functions: “(1) acquiring, deploying and maintaining nursing resources, (2) transforming nursing resources into nursing services, and (3) producing positive changes in a patient's condition as a result of providing nursing services” ([15]; p. 6). These three subsystems are operationalized through 14 dimensions and 51 indicators. The NCPF is the result of an extensive survey of the literature on nursing measurement models and performance indicators. The authors were inspired by the following: (1) the work of Donabedian [16]; (2) Parsons' [17] social systems analysis; and (3) Bertalanffy's [18] systems theory. These works led them to propose a comprehensive and integrative model of performance consisting of indicators that are potentially nursing-sensitive. The article by Dubois et al. [15] clarifies in detail the subdimensions, dimensions, and indicators.

As such, these authors define nursing performance as “the capacity demonstrated by an organization or an organizational unit to acquire the needed nursing resources and use them in a sustainable manner to produce nursing services that effectively improve patients' conditions” ([15]; p. 6).

## 3. Method

The method used for this study was a scoping review, which is an interpretive survey of the literature. This method took into account the constraints associated with the study's context, that is, the limited development of research and the lack of consensus on performance indicators for ambulatory care nursing. It fostered the iterative inclusion of studies based on their relevance, credibility, and contribution [19, 20]. This method can be used to clarify a complex concept or as a preliminary step before a systematic literature analysis, to assess, for example, whether primary studies are sufficient or whether others might be necessary [20].

**Figure 2:**
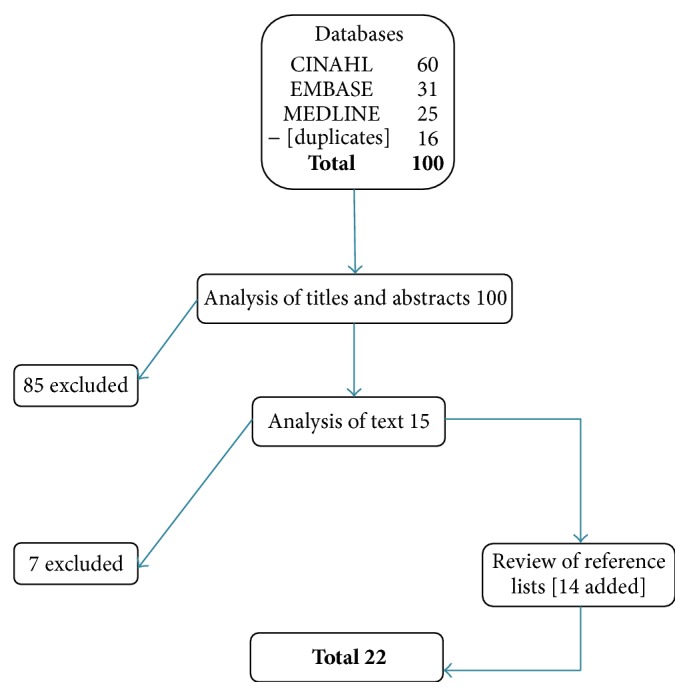
Data extraction and analysis.

A variety of databases were consulted for the literature review, including CINAHL, MEDLINE, and EMBASE, using the following descriptors:* nurse*;* ambulatory-outpatient service*;* clinical quality-performance indicators*;* performance measurement systems*;* minimum data set*;* report cards*. Articles written in English or French between 2000 and 2013 were included, to follow up on the ANA [5], which covered the period from 1980 to 2000. This strategy identified 100 articles that initially appeared relevant. These articles were analyzed in three stages. First, titles and abstracts were reviewed and nonrelevant articles (*n* = 85) were excluded. In-depth analysis of the remaining 15 articles eliminated another group (*n* = 7). Lastly, 14 more articles were identified through snowballing, bringing the final number of relevant articles to 22 (Figure 2). The selected studies, presented alphabetically by author, are as follows:

  Barkauskas et al. [21].
  Bostick et al. [7].
  Chin et al. [22].
  Cohen et al. [23].
  Griffin and Swan [24].
  Griffiths et al. [25].
  Griffiths et al. [26].
  Kedrowski and Weiner [27].
  Levitt et al. [28].
  Mastal [29].
  Mastal [30].
  Matutina et al. [31].
  Morris et al. [32].
  Oermann et al. [33].
  Pitkaaho et al. [34].
  Robinson [35].
  Sawyer et al. [36].
  Shield et al. [37].
  Speros [38].
  Swan [2].
  Swan et al. [3].
  VanDeVelde-Coke et al. [39].

The articles were analyzed using a grid; this analysis compiled data on the research designs used, the scientific criteria applied, the presence or absence of a conceptual model, and especially the performance indicators mentioned in each article. This grid was used for two processes: (1) to search the articles for the indicators already identified by Dubois et al. [15] and count the number of times each was mentioned, and (2) to identify indicators that were not in the framework of Dubois et al. [15] and that could theoretically be specific to ambulatory care nursing.

## 4. Results

The first observation of note is that the number of articles on ambulatory care nursing performance is very small: 22 articles is not many, especially since several presented significant methodological shortcomings and five provided almost no information on their methodology. The research strategies varied: 10 descriptive studies, six literature surveys, five developmental studies (e.g., development of measurement scales), and one longitudinal study. Of the 22 articles, 13 looked at indicators in ambulatory settings in general and nine looked at specific settings such as mental health (*n* = 2) and primary care (*n* = 7) (private medical practice, nurse-managed centre). Only four articles used a model or a classification structure to categorize indicators (e.g., structure-process-outcomes).

The objective of our scoping review was to identify performance indicators that would be sensitive to ambulatory care nursing. The first question was whether the NCPF indicators identified in acute care were found in our scoping review on ambulatory care. The results showed that all the indicators identified in the NCPF were indeed also found in the articles examined in our survey and thus appeared to apply to ambulatory care. A second question was whether our survey identified any other indicators that would be specific to ambulatory care. The results showed that no other indicators were added that were specific to ambulatory care. On the other hand, the survey brought greater detail to two of the NCPF indicators and added three indicators that were missing from that framework:* equity* [28, 37] and* accessibility* [22, 23, 27, 28, 33, 36, 37] as perceived by the patient and* health status* [2, 3, 23, 25, 30, 35, 36], which includes a variety of physiological markers. With regard to the two indicators that were described in more detail, the first was the nursing intervention that involves interacting with the patient [29, 33, 36, 37], which is not detailed as such in the NCPF, but was very much present in the articles on ambulatory care. The second had to do with support to practice, which involves providing nurses with guidelines or directives [23, 24, 27, 28, 35, 37].

The results show considerable variation in how many times the indicators are mentioned in the acute care literature versus in the ambulatory care literature. In ambulatory care, the five indicators mentioned most often were as follows: “promotion/prevention” [2, 3, 7, 21–24, 26–28, 30–33, 35, 36, 39], “management of problems and symptoms” [2, 22–26, 28, 29, 32, 35–39], “assessment, planning, and evaluation” [2, 7, 21, 22, 24, 28–30, 32, 35–39], “ability to achieve appropriate self-care” [2, 3, 21–23, 26–28, 30, 32, 33, 36, 39], and “types of nursing staff supply” [2, 3, 7, 21–39]. In acute care, the predominant indicators were in two groups: “nursing staff supply” and “risk outcomes and safety,” mainly in relation to falls, medication administration errors, pressure ulcers, and nosocomial infections.  Table 1 presents the differences between ambulatory and acute care with regard to the indicators related to the three NCPF subsystems.

These differences are expressed in terms of the three subsystems. In fact, 20.4% of the mentions regarding performance indicators in our study and 28.8% in Dubois et al. [15] fell within the subsystem “acquiring, deploying, and maintaining resources.” For the “transforming resources into services” subsystem, those percentages were 41.1% in our study and 17.6% in Dubois et al. [15], and for the subsystem “producing changes in patients' conditions,” they were 38.6% in our study and 53.6% in Dubois et al. [15].

## 5. Discussion

The theoretical framework of Dubois et al. [15] provides a solid foundation based on a systematic literature review. That framework brings together indicators used to measure nursing performance based on a systematic approach in which three subsystems are interrelated.

The results of this study show that all the indicators already proposed in the NCPF apply to ambulatory care and that the five indicators identified in our survey can be added to the NCPF. These five indicators are not specific to ambulatory care. This result is of interest because our initial assumption was that this study would identify indicators to measure nursing performance specifically in ambulatory care. However, the indicators found in the literature appeared rather to be generic and applicable to different settings, whether acute care, long-term care, or ambulatory services. With regard to the three new indicators added to the NCPF—equity, accessibility, and health status—the first two are important in ambulatory care, particularly from the patient's perspective (e.g., wait times for triage or nursing consultation). Health status was placed in the group “joint contribution of nursing and other systems” and presents very diverse measures, such as haemoglobin A1C, LDL cholesterol level, asthma, and blood pressure. These are very specific measures that are not interrelated and whose nursing sensitivities are quite varied. As such, the health status indicator might be considered a construct. With regard to the two indicators that were made more specific (i.e., nurse-patient interaction and support to practice), they shed different light on the topic by taking the care providers' perspective, which would seem to be important in the ambulatory setting.

The differences between the ambulatory care and acute care literatures appeared rather to lie in the frequency with which various indicators were used. In fact, the choice of performance indicators seemed to be related to the prevailing practices in each setting as well as to the quality and safety issues in the different settings. For instance, indicators selected in the ambulatory setting were more often related to the “transforming resources into services” subsystem: “promotion/prevention,” “management of problems and symptoms,” and “assessment, planning, and prevention.” Indicators related to “patient empowerment” were also well developed in ambulatory care, whereas in acute care, indicators related to “risk outcomes and safety” and “nursing staff supply” were used much more often to measure performance.

The indicators that predominate in ambulatory care clearly reflect the nature of nursing interventions and concerns, in which they highlight nurses' contributions to patients and their families. Moreover, indicators such as “patient/family involvement,” “continuity,” “responsiveness,” and “patient satisfaction/complaints” clearly reflect nurses' role in ambulatory care, given the objectives aimed at implementing a patient and family-centred approach. Other indicators are infrequently cited but remain important in this context, such as “accessibility” and “scope of practice.” In fact, ensuring that nursing consultations and emergency room triage are accessible to patients is a key nursing concern, especially since there seems to be considerable variation in the scope of nursing practice in ambulatory services.

Given the diversity of ambulatory settings, it would be difficult to define a single set of indicators that could be applied universally to emergency services, telehealth services, dialysis, postpartum care, diabetic patients, and so forth. Moreover, organizational characteristics can also influence practices and ultimately care outcomes; such characteristics include team structure, the care setting, the practice environment, and the length of treatment. On the other hand, there are certain practices that are common to all settings regardless of context, such as promotion and prevention, management of problems and symptoms, continuity, and responding to the needs of patients and their families.

As such, based on our research we are able to propose a theoretical framework for the evaluation of nursing performance that can be applied in both acute and ambulatory care. As mentioned in the introduction, there is currently very little available evidence on nursing performance in ambulatory care. This study provides managers with an important tool to remedy this situation by presenting a comprehensive overview of indicators that can be used in evaluating ambulatory care. In this respect, the expert consensus report prepared by Martinez et al. [6] is complementary to our approach, as the objective of those authors was to identify a limited number of indicators.

Using indicators to evaluate nursing performance is, in itself, a complex exercise that presents challenges in terms of feasibility. The proposed framework encompasses all possible indicators. However, in practice only a limited number of indicators should be selected, and these need to make sense for the health professionals and enable a certain amount of benchmarking [13, 15]. The use of indicators, even if only a few, is a first step in the rigorous examination of care quality and safety and serves as a starting point for practice improvement.

## 6. Limitations

One limitation of this study is that the evidence presented in most of the selected articles was not very strong. Also, the process of categorizing indicators was sometimes complicated because (1) some articles were muddled about the definition of terms, such as domain, indicator, and measure, (2) indicators were often formulated differently from one article to another, and (3) indicators were often not explicitly defined.

## 7. Conclusion

This study has extended the theoretical framework of Dubois et al. [15] to encompass ambulatory care nursing. It provides a more recent synthesis of the state of current knowledge on performance indicators for ambulatory care nursing. It enhances the NCPF with the addition of five new indicators.

However, much remains to be done in terms of developing measures for these indicators and setting up systems for managing performance in ambulatory care nursing. Finally, while it is essential to know what indicators are nursing-sensitive, it is equally essential to incorporate these into processes for evaluating interprofessional performance.

## Figures and Tables

**Table 1:** Comparison of indicators from this scoping review and those of Dubois et al. [15].

	Indicators		Indicators	
	Scoping review		Dubois et al. [15]	
	(*n* ^*∗*^ = 319)		(*n* = 444)	
	*n*	%	*n*	%
Acquiring, deploying, and maintaining resources				
Nursing staff supply				
Quantity	7	2.2	17	3.8
Types	14	4.4	18	4.1
Skill mix	9	2.8	18	4.1
Patient profiles	2	0.6	12	2.7
Management of working conditions				
Physical, material, and technological resources	7	2.2	10	2.3
Employment conditions	6	1.9	13	2.9
Nursing staff maintenance				
Health and quality of life at work	1	0.3	2	0.5
Satisfaction with working conditions	5	1.6	9	2.0
Retention	4	1.3	9	2.0
Absenteeism	3	0.9	6	1.4
Economic sustainability				
Costs	6	1.9	3	0.7
Costs per visit	1	0.3	11	2.5
Total: acquiring, deploying, and maintaining resources	65	20.4	128	28.8

Transforming resources into services				
Nursing processes	3	0.9	NA	NA
Assessment, planning, and evaluation	14	4.4	9	2.0
Management of problems and symptoms	14	4.4	13	2.9
Promotion/prevention	17	5.3	7	1.6
Hospital-community integration	6	1.9	4	0.9
Scope of practice	2	0.6	3	0.7
Nurse-patient interaction	4	1.3	NA	NA
Patient experience	4	1.3	NA	NA
Continuity	11	3.4	8	1.8
Patient/family involvement	12	3.8	13	2.9
Responsiveness	11	3.4	4	0.9
Equity	2	0.6	NA	NA
Accessibility	7	2.2	NA	NA
Practice environment	14	3.4	12	2.7
Support to practice (e.g., guidelines and directives)	6	1.9	NA	NA
Professional satisfaction	4	1.3	5	1.1
Total: transforming resources into services	131	41.1	78	17.6

Producing changes in patients' conditions				
Risk outcomes and safety	4	1.3	NA	NA
Falls	4	1.3	17	3.8
Injuries	3	0.9	6	1.4
Medication management: errors	4	1.3	14	3.2
Pulmonary infections	2	0.6	5	1.1
Pressure ulcers/skin integrity	4	1.3	17	3.8
Urinary infections	2	0.6	6	1.4
Intravenous infections	—	—	7	1.6
Abuses	1	0.3	3	0.7
Nosocomial infections	1	0.3	12	2.7
Failure to rescue	1	0.3	4	0.9
Patient comfort and quality of life				
Hygiene	2	0.6	4	0.9
Physical and chemical restraints	—	—	8	1.8
Management of symptoms	11	3.4	22	5.0
Incontinence	4	1.3	5	1.1
Comfort and quality of life	6	1.9	3	0.7
Patient empowerment				
Ability to achieve appropriate self-care	13	4.1	11	2.5
Adoption of health-promoting behaviours	8	2.5	2	0.5
Patient functional status	2	0.6	NA	NA
Physical functional capacity	8	2.5	17	3.8
Cognitive and psychosocial functional capacity	7	2.2	15	3.4
Functional capacity	5	1.6	3	0.7
Recovery of initial health status	1	0.3	3	0.7
Nutritional status	3	0.9	6	1.4
Patient satisfaction/complaints	13	4.1	17	3.8
Joint contribution of nursing and other systems				
Health status	7	2.2	NA	NA
Readmission	4	1.3	6	1.4
Length of stay	—	—	9	2.0
Complications	2	0.6	7	1.6
Mortality	1	0.3	9	2.0
Total: producing changes in patients' conditions	123	38.6	238	53.6

^*∗*^Total number of indicators in the 22 documents selected.

## References

[1] Seematter-Bagnoud L., Junod J., Jaccard Ruedin H., Roth M., Foletti C., Santos-Eggimann B. (2008). *Offre et recours aux soins médicaux ambulatoires en Suisse—projections à l'horizon 2030*.

[2] Swan B. A. (2008). Making nursing-sensitive quality indicators real in ambulatory care. *Nursing Economics*.

[3] Swan B. A., Conway-Phillips R., Griffin K. F. (2006). Demonstrating the value of the RN in ambulatory care. *Nursing Economics*.

[4] Pitman N. J. (2010). *Scope and Standards of Practice for Professional Ambulatory Care Nursing*.

[5] ANA (2000). *Nursing Quality Indicators Beyond Acute Care: Literature Review*.

[6] Martinez K., Battaglia R., Start R., Mastal M. F., Matlock A. M. (2015). Nursing-sensitive indicators in ambulatory care. *Nursing Economic$*.

[7] Bostick J. E., Riggs C. J., Rantz M. J. (2003). Quality measurement in nursing: an update of where we are now. *Journal of Nursing Care Quality*.

[8] Conway-Phillips R. (2006). Ambulatory care nurses speak out: the value of ambulatory care nurses in the workplace. *AAACN Viewpoint*.

[9] Haas S., Swan B. A., Haynes T. (2013). Developing ambulatory care registered nurse competencies for care coordination and transition management. *Nursing Economics*.

[10] Gandhi T. K., Weingart S. N., Borus J. (2003). Adverse drug events in ambulatory care. *The New England Journal of Medicine*.

[11] Gurwitz J. H., Field T. S., Harrold L. R. (2003). Incidence and preventability of adverse drug events among older persons in the ambulatory setting. *The Journal of the American Medical Association*.

[12] Webster J. S., King H. B., Toomey L. M., Henriksen D. K., Battles J. B., Keyes M. A., Grady M. L. (2008). Understanding quality and safety problems in the ambulatory environment: seeking improvement with promising teamwork tools and strategies. *Advances in Patient Safety: New Directions and Alternative Approaches, Volume 3: Performance and Tools*.

[13] Doran D., Mildon B., Clarke S. P. Vers un bulletin national de la pratique infirmière: Synthèse des connaissances. http://www.nhsru.com/wp-content/uploads/8083781_FR_Knowledge_Synthesis_Toward_a_National_Nursing_Report_Card_March_111.pdf.

[14] Swan B. A., Haas S. A., Chow M. (2010). Ambulatory care registered nurse performance measurement. *Nursing Economics*.

[15] Dubois C.-A., D'Amour D., Pomey M.-P., Girard F., Brault I. (2013). Conceptualizing performance of nursing care as a prerequisite for better measurement: a systematic and interpretive review. *BMC Nursing*.

[16] Donabedian A. (1988). The quality of care. How can it be assessed?. *Journal of the American Medical Association*.

[17] Parsons T. (1960). *Structure and Process in Modern Societies*.

[18] Bertalanffy V. (1968). *General System Theory*.

[19] Davis K., Drey N., Gould D. (2009). What are scoping studies? A review of the nursing literature. *International Journal of Nursing Studies*.

[20] Levac D., Colquhoun H., O'Brien K. K. (2010). Scoping studies: advancing the methodology. *Implementation Science*.

[21] Barkauskas V. H., Pohl J. M., Benkert R., Wells M. A. (2005). Measuring quality in nurse-managed centers using HEDIS measures. *Journal for Healthcare Quality*.

[22] Chin W. Y., Lam C. L. K., Lo S. V. (2011). Quality of care of nurse-led and allied health personnel-led primary care clinics. *Hong Kong Medical Journal*.

[23] Cohen J., Saylor C., Holzemer W. L., Gorenberg B. (2000). Linking nursing care interventions with client outcomes: a community-based application of an outcomes model. *Journal of Nursing Care Quality*.

[24] Griffin K. F., Swan B. A. (2006). Linking nursing workload and performance indicators in ambulatory care. *Nursing Economics*.

[25] Griffiths P., Murrells T., Maben J., Jones S., Ashworth M. (2010). Nurse staffing and quality of care in UK general practice: cross-sectional study using routinely collected data. *The British Journal of General Practice*.

[26] Griffiths P., Richardson A., Blackwell R. (2012). Outcomes sensitive to nursing service quality in ambulatory cancer chemotherapy: systematic scoping review. *European Journal of Oncology Nursing*.

[27] Kedrowski S. M., Weiner C. (2003). Performance measures in ambulatory care. *Nursing Economics*.

[28] Levitt C., Chen X., Hilts L., Dolovich L., Price D., Kalpana N. (2013). Developing an institute of medicine-aligned framework for categorizing primary care indicators for quality assessment. *Healthcare Quarterly*.

[29] Mastal P. (2000). Report cards: proving the value of ambulatory care nursing. *AAACN Viewpoint*.

[30] Mastal P. (2001). Ambulatory nursing outcomes. *AAACN Viewpoint*.

[31] Matutina R. E., Hamner S. B., Battaglia R. (2012). Redefining and categorizing the perceived value of the RN in ambulatory care. *AAACN Viewpoint*.

[32] Morris R., MacNeela P., Scott A. (2010). The Irish nursing minimum data set for mental health—a valid and reliable tool for the collection of standardised nursing data. *Journal of Clinical Nursing*.

[33] Oermann M. H., Dillon S. L., Templin T. (2000). Indicators of quality of care in clinics: patients' perspectives. *Journal for Healthcare Quality*.

[34] Pitkaaho T., Partanen P., Vehvilainen-Julkunen K., Miettinen M. (2009). Identification and usability of data-based nurse staffing indicators: a pilot study in Kuopio University Hospital. *Studies in Health Technology & Informatics*.

[35] Robinson J. (2001). *Core Curriculum for Ambulatory Care Nursing*.

[36] Sawyer L. M., Berkowitz B., Haber J. E. (2002). Expanding American Nurses Association nursing quality indicators to community-based practices. *Outcomes Management*.

[37] Shield T., Campbell S., Rogers A., Worrall A., Chew-Graham C., Gask L. (2003). Quality indicators for primary care mental health services. *Quality and Safety in Health Care*.

[38] Speros C. I. A system to evaluate provider performance within the Shelby County healthcare network. http://search.ebscohost.com/login.aspx?direct=true&db=rzh&AN=2003050741&lang=fr&site=ehost-live.

[39] VanDeVelde-Coke S., Doran D., Grinspun D. (2012). Measuring outcomes of nursing care, improving the health of Canadians: NNQR (C), C-HOBIC and NQuiRE. *Nursing Leadership*.

